# Viability assessment of the liver during ex-situ machine perfusion prior to transplantation

**DOI:** 10.1097/MOT.0000000000001152

**Published:** 2024-05-17

**Authors:** Puck C. Groen, Otto B. van Leeuwen, Jeroen de Jonge, Robert J. Porte

**Affiliations:** Department of Surgery, Division of Hepato-Pancreato- Biliary and Transplant Surgery, Erasmus MC Transplant Institute, University Medical Center Rotterdam, Rotterdam, the Netherlands

**Keywords:** ex-situ machine perfusion, liver transplantation, liver viability assessment

## Abstract

**Purpose of review:**

In an attempt to reduce waiting list mortality in liver transplantation, less-than-ideal quality donor livers from extended criteria donors are increasingly accepted. Predicting the outcome of these organs remains a challenge. Machine perfusion provides the unique possibility to assess donor liver viability pretransplantation and predict postreperfusion organ function.

**Recent findings:**

Assessing liver viability during hypothermic machine perfusion remains challenging, as the liver is not metabolically active. Nevertheless, the levels of flavin mononucleotide, transaminases, lactate dehydrogenase, glucose and pH in the perfusate have proven to be predictors of liver viability. During normothermic machine perfusion, the liver is metabolically active and in addition to the perfusate levels of pH, transaminases, glucose and lactate, the production of bile is a crucial criterion for hepatocyte viability. Cholangiocyte viability can be determined by analyzing bile composition. The differences between perfusate and bile levels of pH, bicarbonate and glucose are good predictors of freedom from ischemic cholangiopathy.

**Summary:**

Although consensus is lacking regarding precise cut-off values during machine perfusion, there is general consensus on the importance of evaluating both hepatocyte and cholangiocyte compartments. The challenge is to reach consensus for increased organ utilization, while at the same time pushing the boundaries by expanding the possibilities for viability testing.

## INTRODUCTION

Liver transplantation is a lifesaving treatment for patients with end-stage liver disease, acute liver failure and primary liver cancer. Unfortunately, with increasing rates of liver disease, the number of transplantable organs available is unable to meet demand, and mortality on the waiting list is high [1]. The shortage of donor livers, in combination with altering organ donor demographics, such as ageing population and increased obesity, has led to the increased utilization of livers from extended criteria donors (ECD) of suboptimal quality [2,3]. ECD livers provide an increased risk of developing early allograft dysfunction (EAD), primary nonfunction (PNF), or posttransplant ischemic cholangiopathy [4–11]. Predicting the postreperfusion viability of ECD livers prior to transplantation is one of the most challenging calls to make in organ transplantation. For recipient safety reasons, high-risk donor livers are, therefore, often declined for transplantation [12].

Ex-situ machine perfusion now provides a unique opportunity to assess ECD liver function before transplantation. Viability assessment has mainly been described during normothermic machine perfusion (NMP). However, there are new approaches for assessment during hypothermic machine perfusion (HMP). To add value to known donor and recipient risk factors for worse posttransplant outcome, reliable criteria to determine donor liver viability and predict save use need to be established. This article reviews currently used and promising new viability criteria during ex-situ liver machine perfusion. 

**Box 1:**
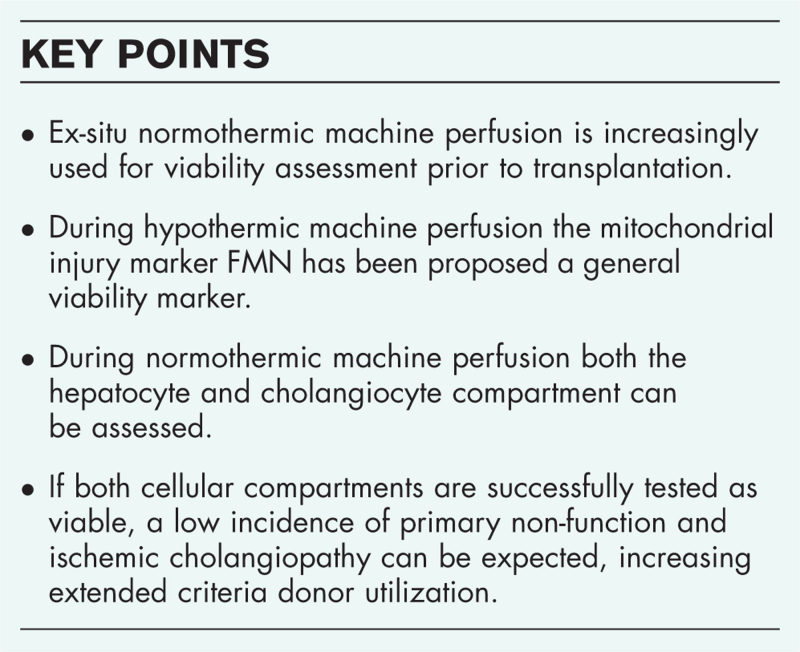
no caption available

## VIABILITY ASSESSMENT

**FIGURE 1:**
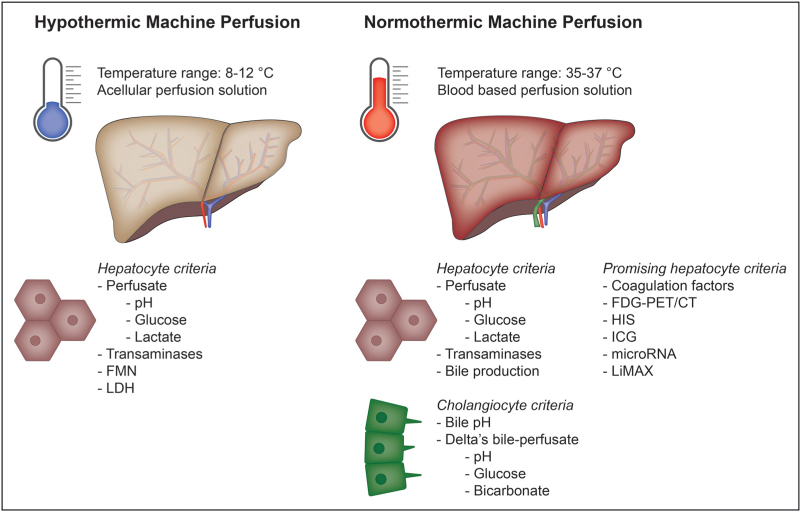
Viability criteria during hypothermic and normothermic machine perfusion. FDG, 18F-fluorodeoxyglucose; FMN, flavin mononucleotide; HIS, hyperspectral imaging; ICG, indocyanine green; LDH, lactate dehydrogenase; LiMAx, liver maximum capacity.

For long-term graft survival, both liver synthetic and detoxification functions and biliary integrity have to be preserved. Therefore, viability criteria have been chosen to test the hepatocyte and cholangiocyte compartment. Most viability criteria can only be assessed during NMP, but some tests are also possible during HMP (Fig. 1). Table 1 provides an overview of the viability criteria currently used in clinical practice, which are further discussed below.

**Table 1:** Viability criteria used in the clinical practice

Reference	Number of livers	DCD/DBD	Viability criteria	Outcomes
HMP				
Eden *et al.*[16] (2023)	Perfused: not specified Transplanted: 158	Not specified	FMN at 30 min of HOPE (<6000 A.U.) NADH at 30 min of HOPE (<8.000 A.U.)	89% 1-year graft survival 7 PNF 11 IC 53 AS 9 bile leakage
Patrono *et al.*[17] (2020)	Perfused: 50 Transplanted: 50	0/50	Perfusate lactate, AST, ALT, LDH, glucose, and pH during DHOPE	1 graft loss 13 EAD
Schlegel *et al.*[15] (2020)	Perfused: 50 Transplanted: 50	32/18	Perfusate, tissue and mitochondria during HOPE: FMN at 30 min (<8000 A.U.) NADH (<10 000 A.U.)	7 graft loss (unspecified)
Muller *et al.*[14] (2019)	Perfused: 54 Transplanted: 54	35/19	FMN within 30 min of HOPE	7 graft loss 4 PNF 1 IC
NMP				
Van Leeuwen *et al.*[33] (2022)	Perfused: 54 Transplanted: 34	53/1	After 2.5 h of NMP - Lactate <1.7 mmol/l - Perfusate pH 7.35–7.45 - Bile production >10 ml, of which ≥4 ml in the last hour - Bile pH > 7.45 - Delta pH bile and perfusate >0.10 - Delta sodium bicarbonate bile and perfusate >5.0 - Delta glucose bile and perfusate <−5.0	94% 1-year graft survival 1 NAS 12 AS 4 bile leakage
Seidita *et al.*[31] (2022)	Perfused: 19 Transplanted: 17	3/16	- Lactate clearance normalization or at least halving of lactates at end of perfusion - pH > 7.3 - Bile production - Vascular flow HA and PV	94% 1-year graft survival 1 EAD
Quintini *et al.*[29] (2022)	Perfused: 21 Transplanted: 15	13/8	Within 6 h of NMP, ≥2 of the following - Perfusate lactate <4.5 mmol/l or decrease of 60% from peak in first 4 h - Bile production >2 mL/h - Stable HA and PV flow (>0.05 ml/min/g and >0.4 ml/min/g) - Macroscopic homogenous perfusion and soft consistency	7 EAD 1 IC
Zhang *et al.*[36] (2020)	Perfused: 4 Transplanted: 4 Retrospect	3/1	Within 4 h of NMP - Perfusate lactate <2.5 mmol/l - Bile production - Stable HA and PV flow (>150 ml/min and >500 ml/min) - Perfusate pH > 7.3	100% 6-months graft survival 1 EAD 1 AS
Reiling *et al.*[30] (2020)	Perfused: 10 Transplanted: 10	5/5	After 4 h of NMP - Perfusate lactate <2 mmol/l within 2 h - Metabolism of glucose, evidenced by decreasing trend by 4 h - Physiological pH without continuous need for sodium bicarbonate - Stable HA and PV flows - Homogenous graft perfusion with soft parenchyma consistency - Bile production (no lower limit)	100% 6-months graft survival 5 EAD 1 AS 1 anastomotic leak
Mergental *et al.*[25] (2020)	Perfused: 31 Transplanted: 22	14/17	Within 4 h of NMP - Perfusate lactate ≤2.5 mmol/l And ≥2 of the following criteria - Evidence of bile production - Perfusate pH ≥7.30 - Metabolism of glucose - Stable HA and PV flows (≥150 ml/min and ≥500 ml/min) - Homogenous perfusion with soft consistency of the parenchyma	86.4% 1-year graft survival 7 EAD 4 NAS 2 AS
Cardini *et al.*[22] (2020)	Perfused: 34 Transplanted: 25	4/30	After 2 h of NMP - Rapid decrease and maintenance of lactate levels (first 2 h of NMP) - Bile output and biliary pH - Maintaining a physiological perfusate pH without sodium bicarbonate - Warning signals: exceptionally high or sharp incline of AST, ALT and LDH	88% graft survival at 20 months 7 AS 3 bile leakage
Bral *et al.*[21] (2019)	Perfused: 46 Transplanted: 43	10/33	- Lactate level at start perfusion - Lactate clearance - Necessity of bicarbonate pH correction - Bile production	100% 3-month graft survival 11 EAD 2 NAS 6 AS
Watson *et al.*[35] (2018)	Perfused: 47 Transplanted: 22	35/12	- Peak lactate fall ≥4.4 mmol/l/kg/h - ALT <600 iU/l at 2 h - Perfusate pH > 7.2 with ≤30 mmol/l bicarbonate supplementation - Maximum bile pH > 7.5 - Bile glucose concentration ≤3 mmol/l or ≥10 mmol less than perfusate glucose - Falling glucose beyond 2 h or perfusate glucose under 10 mmol/l with subsequent fall during challenge with 2.5 g glucose	1 PNF 1 EAD 4 IC

ALT, alanine transaminase; AS, anastomotic biliary strictures; AST, aspartate aminotransferase; DBD, donation after brain death; DCD, donation after circulatory death; DHOPE, dual hypothermic oxygenated machine perfusion; EAD, early allograft dysfunction; HA, hepatic artery; HMP, hypothermic machine perfusion; HOPE, hypothermic oxygenated machine perfusion; IC, ischemic cholangiopathy; LDH, lactate dehydrogenase; NADH, nicotine adenine dinucleotide reduced; NAS, nonanastomotic biliary strictures; NMP, normothermic machine perfusion; PNF, primary nonfunction; PV, portal vein.

## VIABILITY ASSESSMENT DURING HYPOTHERMIC MACHINE PERFUSION

At 4–10 °C, metabolic activity is significantly reduced to 6–15% of normal, posing challenges for real-time assessment of metabolism and function [13]. Nevertheless, several perfusate analyses can be performed at 7–10 °C, at which HMP is typically performed.

### Flavin mononucleotide

Flavin mononucleotide (FMN) can be assessed during both NMP and HMP. FMN, typically bound to mitochondrial complex I under physiological conditions, is released from damaged complex I during ischemia and subsequent reoxygenation. The Zurich group analyzed FMN in 54 livers during oxygenated-HMP and demonstrated a strong correlation between FMN release, coagulation factors levels and peak transaminases post-transplantation, and showed predictive value for EAD [14]. In 2020, the group form Zurich measured FMN in the perfusate of 50 livers, with elevated concentrations of FMN in the perfusate correlating with a high rate of graft loss [15]. The authors advise against transplantation if FMN concentration exceeds 8000AU after 30 minutes of oxygenated-HMP. In their most recent work, which includes all perfusions from previous work, they suggest a FMN threshold of 6000AU and a NADH threshold of 8000AU after 30 minutes of perfusion [16]. Confirmation of these single center observations in other centers is eagerly awaited.

### Perfusate analysis

Patrono *et al.*[17] have analyzed perfusate in 50 DBD livers during oxygenated-HMP and assessed levels of aspartate aminotransferase (AST), alanine transaminase (ALT), lactate dehydrogenase (LDH), glucose, lactate and pH at 90 min of perfusion in relation to postoperative outcomes [17]. All parameters, except lactate, correlated with EAD. However, macrovesicular steatosis was the only independent predictor of EAD, as all perfusate parameters were closely correlated to the severity of steatosis.

## VIABILITY ASSESSMENT DURING NORMOTHERMIC MACHINE PERFUSION: HEPATOCYTE VIABILITY

NMP is typically performed at 35–37 °C, enabling assessment of a metabolically active liver. The criteria for hepatocyte viability are based on perfusate analysis, perfusion parameters and bile production.

### Lactate levels

During NMP, three phases in lactate dynamics can be typically identified; initially increasing, peaking at 1 h, followed by a rapid clearance within 2 h, ending in a stable state characterized by consistently low lactate levels during the remainder of the perfusion [18,19]. Nondecreasing lactate levels in the perfusate are generally considered a sign of poor graft function. As shown in Table 1, all clinical studies incorporated perfusate lactate clearance [20–36]. It is noteworthy that cut-off values range between 1.7 and 4.5 mmol/l, and lactate is measured at different time points during NMP. Watson *et al.*[35] integrated liver weight in the equation for lactate clearance, leading to a more accurate estimation of clearance per gram liver tissue. Seidita *et al.*[31], Cardini *et al.*[22] and Bral *et al.*[21] state that lactate should decrease over time, without further specification. In addition to the varying cut-off values, controversy exists on whether lactate clearance is an accurate marker of graft viability. The Zurich group showed lactate clearance up to 24 h of NMP in four explant cirrhotic livers, questioning the predictive value of lactate clearance in early assessment [37^▪^].

### Glucose levels

The liver is a key metabolic organ with a major role in glucose metabolism, including glycogenolysis, gluconeogenesis and glycolysis [38]. Glycogenolysis, an ATP-independent process, continues during static cold storage (SCS) driven by the lack of ATP, which is demonstrated by increasing glucose levels at the beginning of NMP. In viable livers, high levels of glucose should prevent glycogenolysis and trigger glycogenesis, and as a result, glucose levels will decrease. Normal levels of glucose during NMP can reflect minimal ischemia, but it can also point to glycogen exhaustion or extensive liver injury [35]. A glucose challenge test can rule out liver injury: a viable liver metabolizes glucose and levels in the perfusate will drop [25,39,40]. Only Watson *et al.*[35] included a glucose challenge in their viability criteria. Both Reiling *et al.*[30] and Mergental *et al.*[25] included a decreasing glucose trend.

### Acid–base homeostasis

The liver is an important regulator of acid–base homeostasis. Healthy livers tend to have better pH regulation and stabilization. All groups except Quintini *et al.*[29], have included a near physiological pH, from 7.20 to 7.45, as viability criterion. Watson *et al.*[35] described a NMP procedure of a liver requiring more than 30 mmol/l bicarbonate, much more than any other livers, and this liver developed PNF. Therefore, they incorporated a maximum of 30 mmol/l bicarbonate bolus support. pH levels are influenced by perfusate composition, addition of sodium bicarbonate and partial pressure of CO_2_ and therefore pH is best used in combination with other criteria to determine viability.

### Transaminases

Liver transaminases synthesize and break down amino acids and convert energy storage molecules. Damaged hepatocytes release transaminases into the perfusate because of increased membrane permeability. Perfusate transaminase levels, influenced by factors like donor age, steatosis and ischemia time, are nonspecific and require normalization to liver weight and perfusate volume for accurate assessment [41,42]. AST also rises from hemolysis in the perfusion circuit, and therefore ALT is considered more liver-specific [35,39,43]. As shown in Table 1, only Watson *et al.*[35] stated a cut-off value for ALT of less than 6000 IU/l at 2 h of NMP. Furthermore, Cardini *et al.*[22] stated exceptionally high or sharp increase of AST and ALT, without further specification, as a warning signal.

### Perfusion parameters

In liver perfusion, flow in the hepatic artery and portal vein is determined by perfusion pressure and vascular resistance. Prolonged ischemia can damage the microcirculation of the liver, leading to increased vascular resistance and reduced perfusion at fixed pressure, resulting in impaired function [44,45]. Steatotic livers, with narrower sinusoids, exhibit lower flow, leading to secondary hypoxia and reperfusion injury [46]. Elevated vascular resistance during machine perfusion indicates liver injury and later liver dysfunction [45]. Whereas many groups suggest necessity for stable hepatic artery and portal vein flows [20,28,30,31], some added specific target flows [25–27,29], all livers reached the target flows. Quintini *et al.*[29] further specified target flow per unit liver weight, but even here, all livers met the target flows. This indicates that currently flow measurements alone cannot distinguish between viable and nonviable livers.

### Bile production

Bile production is considered one of the higher levels of liver functions, as it requires considerable ATP content. Bile is produced by hepatocytes, and cholangiocytes lining the bile duct alter the composition of bile. Therefore, bile production is associated with hepatocyte viability, whereas bile composition is associated with cholangiocyte viability. All studies in Table 1 have listed production of bile as a viability marker [21,22,25,29–31,33,35,36], demonstrating consensus on the negative implications of absence of bile production. Only two groups have stated a specific cut-off value for bile production. Quintini *et al.*[29] defined a production of more than 2 ml/h as a minimum, Van Leeuwen *et al.*[33] defined a production of more than 10 ml after 2.5 h of NMP, of which at least 4 ml in the last hour. However, graft loss has been described despite proper bile production, and successful transplantations have been reported without proper bile production [35,36,39,47]. As an example, Zhang *et al.*[36] transplanted four livers that did not meet the Groningen group criteria, yet all showed immediate function. It should be noted that malposition of the biliary drain may lead to false-negative absence of bile production.

## VIABILITY ASSESSMENT DURING NORMOTHERMIC MACHINE PERFUSION: CHOLANGIOCYTE VIABILITY

As cholangiocytes play a crucial role in modifying bile composition by re-absorbing solutes and secreting water and bicarbonate, the viability of the cholangiocytes can be assessed by analyzing the composition of bile.

### Bile composition

Low levels of biliary glucose, high levels of bicarbonate and an alkalotic pH are indications of viable cholangiocytes [24,48]. A biliary pH of more than 7.45 was defined by Cardini *et al.*[22] and Van Leeuwen *et al.*[33]. In the cohort of Watson *et al.*[35], 3 out of 16 transplanted livers were unable to achieve a biliary pH greater than 7.40, and developed ischemic cholangiopathy. In addition to low pH, these livers also had similar glucose levels in bile and perfusate, and a low biliary bicarbonate. Therefore, Watson *et al.*[35] stated that the difference between bile and perfusate glucose should at least be 10 mmol/l, and Van Leeuwen *et al.*[33] suggested a delta of at least 5 mmol/l. In addition to the biliary pH and the delta glucose, Van Leeuwen *et al.*[33] also included a delta pH and a delta bicarbonate. Importantly, the VITTAL trial showed that meeting hepatocellular viability criteria is not sufficient to predict ischemic biliary damage [25]. All transplanted livers met the hepatocyte criteria, but nevertheless, 45% of the recipients developed irregularities in the bile ducts, with 18% requiring retransplantation for ischemic cholangiopathy, suggesting nonviable cholangiocytes and bile ducts, despite viable liver parenchyma.

## PROMISING VIABILITY CRITERIA

In addition, clinically implemented viability criteria, new criteria, such as coagulation factors and imaging techniques have been studied in preclinical setting.

### Coagulation factors

The liver is crucial for regulating coagulation and fibrinolysis [49]. NMP circuits are heparinized, yet production of coagulation factors can serve as a viability marker. The long-term NMP study of Eshmuminov *et al.*[40] revealed significantly higher coagulation factor-V levels in the perfusate of functioning versus nonfunctioning livers at 48 h of perfusion, although this difference disappeared thereafter. Weissenbacher *et al.*[50] showed significantly higher levels of von Willebrand factor antigen in the perfusate of EAD livers. Van den Boom *et al.*[51^▪^] measured the international normalized ratio (INR) during NMP. Addition of fibrinogen and/or polybrene, which neutralizes the anticoagulant effects of heparin, was necessary to measure INR in perfusate samples. INR at 150 min or at the end of NMP did not correlate with hepatocyte viability criteria, suggesting that measuring INR may have added value for determining viability.

### Imaging techniques

Orita *et al.* performed a 18F-fluorodeoxyglucose (FDG)-PET/CT to assess viability in two discarded human livers after 1 week of NMP [52]. With FDG-PET/CT, the glucose metabolism can be followed: glucose is taken up by the GLUT-2-transporter, metabolized into FDG-6P and dephosphorylated back to FDG, which then leaves the cells and returns to the circulation [53]. Homogenous FDG uptake was observed in all livers and therefore indicated intact transport, metabolism, and excretion of glucose. Fodor *et al.*[54] utilized hyperspectral imaging (HIS) to analyze the liver parenchyma, monitoring oxygen saturation levels (StO_2_), tissue hemoglobin index (THI), near-infrared perfusion (NIR), and tissue water indices (TWI). HIS was performed during SCS and at 1, 6, 12 h, and end NMP. During NMP, the StO_2_, THI and NIR perfusion indices significantly increased, whereas the TWI drastically decreased. A significantly higher THI was seen in discarded livers compared with transplanted livers end NMP. Kneifel *et al.* also used HIS during NMP at 1, 2 and 4 h of perfusion and showed that StO_2_ and THI predicted lactate values at 1 and 2 h of NMP, THI also predicted lactate values at 4 h [55^▪^]. Lau *et al.*[56^▪▪^] analyzed the indocyanine green (ICG) plasma-disappearance-rate (PDR) and the ICG-perfusion with a near-infrared camera during 7 days of NMP. The ICG-PDR was significantly higher on day 0 in grafts that survived at least 7 days of NMP. ICG perfusion, the distribution of ICG in the liver during NMP, was significantly different at day 0 between long-surviving and short-surviving grafts.

### MicroRNA

Matton *et al.*[57] investigated microRNAs as sensitive, specific, and stable markers for cell function, stress, and injury during NMP. Elevated miRNA-122 was significantly associated with liver injury, although there was no threshold value identified for irreparable damage [58–60].

### Liver maximum capacity test

Our group employed the liver maximum capacity (LiMAx) test to assess liver function during NMP [61]. This is a clinically validated substrate-based cytochromal breath test, which measures ^13^CO_2_ production. The advantage is that all livers can be subjected to an equivalent dose, making direct comparison between results possible. Schurink *et al.* demonstrated a significant correlation between LiMAx values and lactate clearance, and significant inverse correlation between LiMAx values and histological injury. However, no correlation was found between LiMAx values and microRNA-122 or FMN values in the perfusate.

## DISCUSSION AND FUTURE PERSPECTIVES

In this review, we have summarized the emerging consensus on parameters that indicate liver viability during NMP. Viability assessment during HMP could prove to be an important contribution to the field. During NMP lactate clearance, pH stabilization and bile production are uniformly considered as biomarkers of adequate liver function prior to acceptance. There is also growing agreement that besides hepatocellular criteria, cholangiocyte criteria should be considered to prevent biliary ischemic complications post-transplant. In both domains, no consensus was found on specific cut-off values, which might be hampered by considerable differences in perfusion protocols, acceptance criteria and recipient populations. In most viability assessment studies, livers were transplanted into recipients with relatively low Model for End-Stage Liver Disease (MELD) scores. This raises the question if these tested ECD livers are equally suitable for recipients that require a retransplantation and recipients with high MELD scores, as these patients are more at risk for complications and graft loss. So far, recipient factors have not been combined with viability assessment, opposed to the risk scores that were developed recently to balance donor and recipient risks (e.g. UK-DCD score, Balance of Risk score, ET-DRI score).

Considering the differences in length of the viability assessment protocol, the 2.5 h decision moment described by Van Leeuwen *et al.*[33] is swift but proved to be a safe decision moment, without any PNF in their cohort. In their series, about two of three of all tested livers were ultimately transplanted. The short assessment period might, however, be too strict for slowly recovering livers, discarding potentially viable organs meeting the criteria shortly after the decision moment. Other protocols take up to 6 h to reach a decision moment, which might yield a higher ECD liver utilization rate. This indicates that there is potential to further explore the lower limits of organ viability and even push the boundaries more. In addition, organ perfusion and viability assessment can lead to a better understanding of essential hepatobiliary injury and repair processes, opening doors for regenerative medicine applications and potentially repair currently declined organs.

## CONCLUSION

In conclusion, current viability assessment protocols during ex-situ liver perfusion enable well tolerated use of ECD livers. Efforts should be taken to reach consensus on requirements for acceptance to increase wider application of these organs and ultimately reduce waitlist mortality.

## Acknowledgements

*None.*

### Financial support and sponsorship

*None.*

### Conflicts of interest

*There are no conflicts of interest.*

## REFERENCES AND RECOMMENDED READING

Papers of particular interest, published within the annual period of review, have been highlighted as:

▪ of special interest
▪▪ of outstanding interest
